# Membranes with artificial free-volume for biofuel production

**DOI:** 10.1038/ncomms8529

**Published:** 2015-06-24

**Authors:** Nikos Petzetakis, Cara M. Doherty, Aaron W. Thornton, X. Chelsea Chen, Pepa Cotanda, Anita J. Hill, Nitash P. Balsara

**Affiliations:** 1 Department of Chemical & Biomolecular Engineering, University of California, Berkeley, California 94720, USA; 2 CSIRO Manufacturing Private Bag 10, Clayton South, Victoria 3169, Australia; 3 Materials Sciences Division & Environmental Energy Technologies Division, Lawrence Berkeley National Laboratory, Berkeley, California 94720, USA; 4 Joint Center for Artificial Photosynthesis, Lawrence Berkeley National Laboratory, Berkeley, California 94720, USA

## Abstract

Free-volume of polymers governs transport of penetrants through polymeric films. Control over free-volume is thus important for the development of better membranes for a wide variety of applications such as gas separations, pharmaceutical purifications and energy storage. To date, methodologies used to create materials with different amounts of free-volume are based primarily on chemical synthesis of new polymers. Here we report a simple methodology for generating free-volume based on the self-assembly of polyethylene-*b*-polydimethylsiloxane-*b*-polyethylene triblock copolymers. We have used this method to fabricate a series of membranes with identical compositions but with different amounts of free-volume. We use the term artificial free-volume to refer to the additional free-volume created by self-assembly. The effect of artificial free-volume on selective transport through the membranes was tested using butanol/water and ethanol/water mixtures due to their importance in biofuel production. We found that the introduction of artificial free-volume improves both alcohol permeability and selectivity.

## Abstract (web summary)

The free-volume of a polymer is a key parameter in its ability to permit through transport of small molecules. Here, the authors develop a way of introducing different degrees of artificial free-volume to a polymer membrane, and thus tailor its penetrability for applications including biofuel purification.

## Graphical abstract

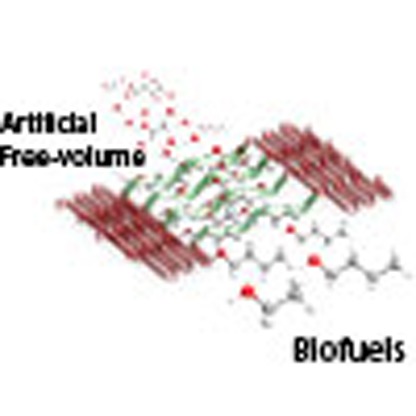

The relationship between free-volume and transport of penetrants through polymer films is exploited in the design of membranes used in a wide variety of processes such as gas separation^1^, pharmaceutical purification^2^ and electrical energy storage^3^,^4^,^5^,^6^. The term free-volume generally refers to disordered voids that occur naturally in polymers with length scales ranging from 0.1 to 1.5 nm. The well-established approach for generating materials with different free-volume is by chemical synthesis of different polymers^1^,^7^,^8^,^9^. For example, the pioneering synthetic work of Masuda and coworkers led to the discovery of poly(1-trimethylsilyl-1-propyne) (PTMSP), which is amongst the most permeable dense materials available to date^1^,^10^. The lack of backbone flexibility and the presence of a bulky side group results in a glassy material with a bimodal distribution of free-volume with cavities sizes centred around *d*=0.6 and *d*=1.4 nm^10^. In contrast, polydimethylsiloxane (PDMS) is a rubbery material with a bimodal distribution of free-volume, but the cavity sizes are centred around *d*=0.4 nm and *d*=0.8 nm. Both materials exhibit reverse selectivity, that is, the transport of bigger molecules is favored over that of smaller molecules^11^. It is generally believed that transport occurs mainly through the larger cavities. Controlling the concentration and size of the larger cavities is thus of considerable importance. The addition of nanoparticles can also influence free-volume due to either the intrinsic properties of the particles or the effect of their presence on the surrounding polymer chains^11^.

The purpose of this paper is to show that it is possible to increase the size and concentration of larger free-volume cavities in rubbery polymers by block copolymer self-assembly. We thus obtain membranes that are chemically identical but contain different levels of free-volume. We study the effect of this free-volume on transport of alcohol/water mixtures through the membrane. Such membranes are needed for cost-effective production of biofuels^12^,^13^.

A major limitation of glassy systems like PTMSP is aging; the efficacy of the membrane deteriorates rapidly during the first few weeks of operation^14^. In an important study, it was demonstrated that the addition of porous aromatic framework particles eliminates physical aging of PTMSP^15^. In contrast, no additives are needed to stabilize the free-volume cavities obtained by block copolymer self-assembly.

## Results

### Membrane fabrication

**Figure 1:**
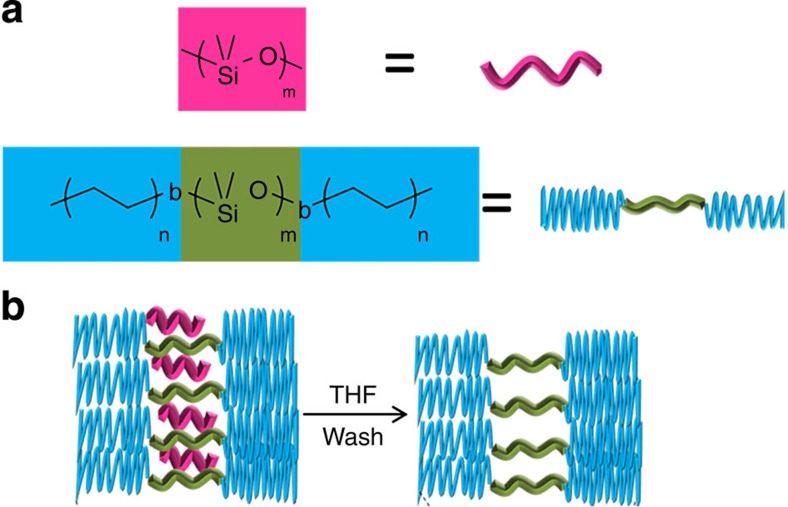
Methodology developed to create artificial free-volume. (**a**) Representation of the homopolymer and block copolymer; (**b**) schematic representation of the formation of artificial free-volume in EDE membranes. The homopolymer is removed by washing out the homopolymer with THF.

Membranes were made by first dissolving a polyethylene-*b*-polydimethylsiloxane-*b*-polyethylene (EDE) triblock copolymer and PDMS homopolymer in cyclohexane, a common solvent for both components at elevated temperatures, and casting a film. The molecular weights of the components and blend composition were adjusted to ensure that the PDMS homopolymer chains were located within the PDMS-rich microdomains of the EDE block copolymer^16^. In the second step, the film was washed with tetrahydrofuran (THF) at room temperature, isolated and dried. This washing results in dissolution and removal of the PDMS homopolymer. The resulting dry film is made up entirely of the EDE copolymer. The volume originally occupied by the PDMS homopolymer in the blended film will be converted into free-volume if the solvent-resistant polyethylene microdomains are not deformed by the processing steps described above. We use the term artificial free-volume to refer to the additional free-volume obtained in the processed and dried film over and above that in PDMS homopolymers. Our approach for generating artificial free-volume is shown schematically in Fig. 1.

The role of the polyethylene block is to prevent relaxation of the PDMS microdomains containing artificial free-volume. It is well-known that macroscopic samples of semicrystalline polyethylene (PE) do not exhibit physical aging. This property, combined with the solvent resistance of PE at room temperature, makes it ideal for the present study. Our approach utilized well-defined high-molecular weight EDE triblock copolymers, which we recently synthesized for the first time^17^, and work by Wong *et al*^16^. In principle, this process could result in polymer films with mesoporosity^18^,^19^,^20^,^21^, that is, pores with radii >2 nm, artificial free-volume, or PDMS microdomains with no additional free-volume.

EDE/PDMS blends were prepared by solvent casting on a temperature-controlled heated stage. Solutions of EDE, PDMS and cyclohexane (0.08 g EDE per ml of cyclohexane) were cast onto a porous teflon substrate (3 M) at 70 °C. The resulting films, with thicknesses in the 20–30 μm range, were dried in a vacuum oven for about 16 h at room temperature. This was followed by drying and annealing the films for 24 h at 130 °C. We define *φ*_PDMS_ as the total PDMS volume fraction in the blended membranes. For reference, we also studied membranes with no added PDMS homopolymer in which case *φ*_PDMS_ is the PDMS volume fraction in the pure EDE copolymer. The fraction of PDMS microdomains occupied by PDMS homopolymer in the blended films, assuming that all of the homopolymer resides within the microdomans is defined as *f*_ADD_. We also define *r* as the ratio of the molecular weight of the PDMS homopolymer to that of the PDMS middle block in the copolymer (the former is 14 kg mol^–1^ for all the blends). Previous studies have demonstrated that *r*-values between 0.1 and 0.3 result in localization of added homopolymers in the targeted microdomains^21^. Our samples are named EDEXX-YY/ZZ where XX is the total number averaged block copolymer molecular weight (Kg mol^−1^), YY is the PDMS volume percentage in the pure block copolymer, and ZZ is 100 × *f*_ADD_. The characteristics of our samples are summarized in Table 1. The blended membranes were immersed in THF for 5 min to dissolve out PDMS homopolymer chains. Then the membranes were immersed in methanol for 5 min. Three such cycles were performed on each membrane, and the films were dried at room temperature either in a fume hood or in a vacuum oven. In all cases, the difference in mass of the films, measured before and after washing and subsequent drying steps, was within experimental error of the mass of homopolymer PDMS added in the blending step (±5%). The complete removal of the homopolymer was confirmed by integration of the ^1^H nuclear magnetic resonance (NMR) spectroscopy signals corresponding to PE and PDMS obtained from solutions of the dried films using deuterated cyclohexane as the solvent at 70 °C (see supplementary Fig. 1). For consistency, the neat EDE samples with no added homopolymer were subjected to the same processing steps as the blended samples.

### X-ray scattering and electron microscopy

**Figure 2:**
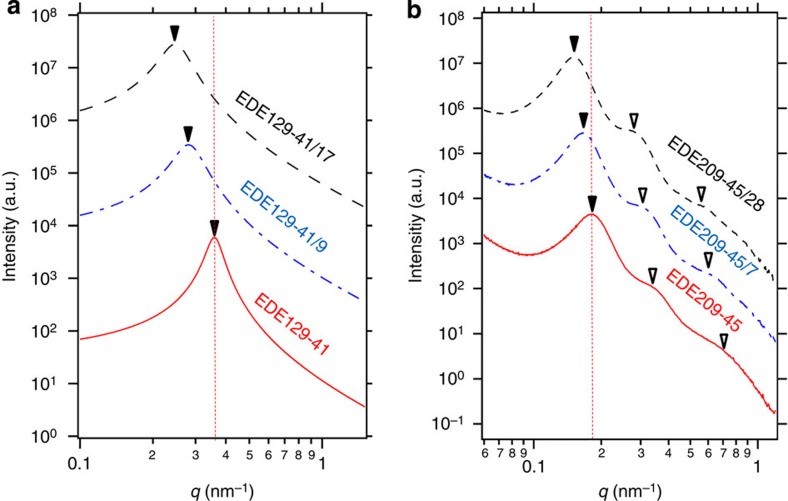
Small-angle X-ray scattering data for the two series of membranes studied. (**a**) EDE129-41; and (**b**) EDE209-45. The locations of the primary peaks are indicated by filled arrows and the locations of the higher-order peaks by hollow arrows. The data collected for the composite membranes were measured after washing away the PDMS homopolymer.

Small angle X-ray scattering (SAXS) measurements were performed on films that were peeled off from the Teflon support. Figure 2 shows background-corrected SAXS profiles for the two series of dried films that we studied. Sample EDE129-41 exhibited a broad primary scattering peak superposed on a monotonically decreasing background, and no higher-order peaks. The centre-to-centre distance between adjacent PDMS microdomains, *d*_EDE_, is estimated to be *d*_EDE_=2*π*/q^*^= 17.4 nm where q* is the magnitude of the scattering vector at the primary scattering peak. The absence of higher-order peaks and the presence of broad primary peaks indicate the presence of a periodic structure with little long-range order. SAXS profiles obtained from samples EDE129-41/9 and EDE129-41/17, the samples with artificial free-volume, were qualitatively similar to EDE129-41, the neat sample (Fig. 2a). However, the creation of artificial free-volume resulted in shifts towards lower q*-values indicating an increase in *d*_EDE_.

**Figure 3:**
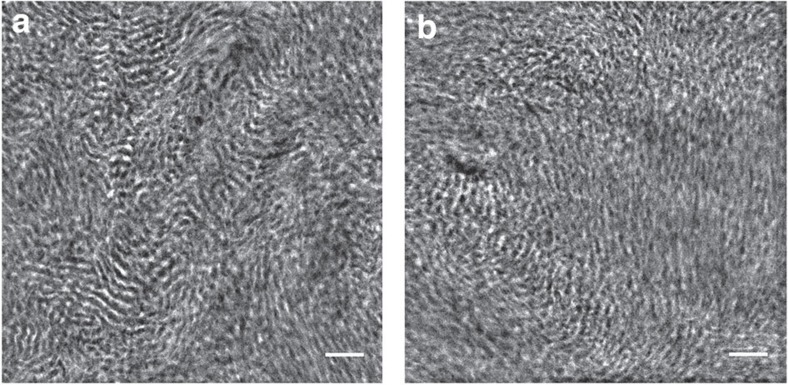
Scanning transmission electron microscopy images of cryo-microtomed samples collected by a high-angle annular dark-field detector. (**a**) Micrograph of membrane EDE129-41 (**b**) Micrograph of membrane EDE129-41/17. Scale bars correspond to 100 nm.

Sample EDE129-41/9 had *d*_EDE_=18.5 nm, and EDE129-41/17 had *d*_EDE_=19.7 nm. If we assume that the sample has a lamellar morphology and that all of the observed increase in *d*_EDE_ occurs in the PDMS microdomains, then the SAXS data indicate expansions of 6.3% and 13.8% in EDE129-41/9 and EDE129-41/17, respectively. This expansion is consistent with our hypothesis of artificial free-volume. SAXS intensity of mesoporous films obtained by templated block copolymer assembly is much higher than that of pure block copolymers due to the increased scattering contrast between mesoporous voids and polymer. The similarity in all of the SAXS profiles shown in Fig. 2a rule out the presence of mesoporous structure in our films. Similar observations were made for the series of samples based on EDE209-45 shown in Fig. 2b. In this case, in addition to the primary scattering peak, higher-order peaks were observed at 2q* and 3q*, consistent with a lamellar morphology. The *d*_EDE_ in this series of membranes increased from *d*_EDE_=32.2 nm for EDE209-45 sample to *d*_EDE_=33.5 nm for EDE209-45/7 sample and *d*_EDE_=34.9 nm for EDE209-45/29 sample, corresponding to an expansion of 4.0% and 8.3%, respectively. (No background subtraction is necessary for the analysis of EDE209-45 samples). Figure 3 shows dark-field transmission electron microscopy images of cryo-microtomed samples of membranes EDE129-41 and EDE129-41/17, following procedures described in the methods section. Both samples exhibit a lamellar morphology with little long-range order, consistent with the SAXS data (Fig. 2a). The dark lamellae represent the polyethylene-rich microdomains, while the bright lamellae represent the PDMS-rich microdomains. The transmission electron microscopy images also rule out the presence of a mesoporous structure.

### Positron annihilation lifetime spectroscopy

The free-volume content of the EDE129-41 series was probed directly by positron annihilation lifetime spectroscopy (PALS). This technique enables determination of the size and relative concentration of free-volume elements by measuring the intensity (*I*_3_) and lifetime (*τ*_3_) of the ortho-positronium states (*o*-Ps)^22^. Our approach for analysing PALS data is described in a study by Merkel *et al.*^22^. The preparation of PALS samples is described in the methods section. The spectra of the PALS signals from all of our samples were consistent with a linear sum of two exponential functions, indicating the presence of free-volume elements with two distinct sizes.

**Figure 4:**
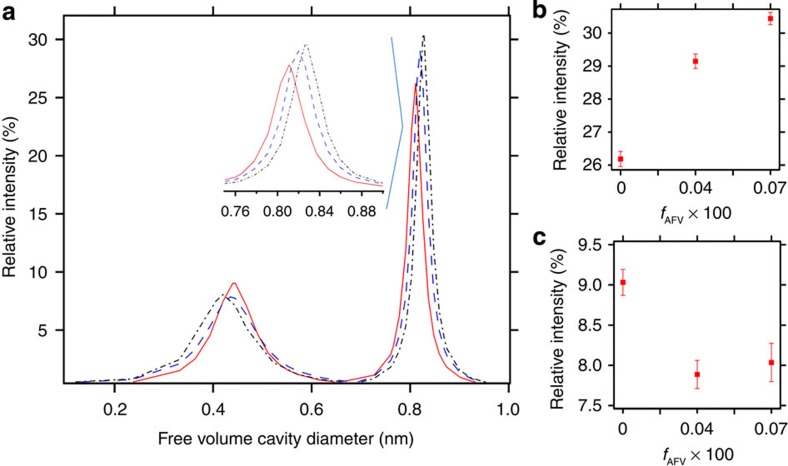
Positron annihilation lifetime spectroscopy (PALS) data for the EDE129-41 series of membranes. (**a**) Free-volume cavity size distributions for membranes EDE129-41, EDE129-41/9 and EDE129-41/17. The inset shows a magnification of the large cavity size population. Solid red line: EDE129-41, dashed blue line: EDE129-41/9, dashed and dotted black line: EDE129-41/17; (**b**) Relative intensity of the large free-volume cavity size population as a function of the amount of homopolymer blended and washed away; (**c**) Relative intensity of the small free-volume cavity size population as a function of the amount of homopolymer blended and washed away. Error bars are calculated by the population s.d. and their values include both experimental as well as the pore size variation within the sample.

Figure 4 shows results of PALS analysis for samples EDE129-41, EDE129-41/9 and EDE129-41/17. The neat EDE129-41 sample exhibits two populations of free-volume elements centred around cavities with diameters of 0.4 and 0.8 nm, respectively. The intensities at the peaks of the distributions corresponding to the small and large cavities are plotted in Fig. 4b and c, respectively. The intensity corresponding to the larger cavities increases with increasing *f*_ADD_, while that corresponding to the smaller cavities decreases with increasing *f*_ADD_. The larger cavities that dominate the distribution functions are shown in Fig. 4a.

The volume of a given type of cavity labelled i, *V*_i_, was calculated by assuming a spherical morphology. The fractional free-volume in our films, FFV, is given by


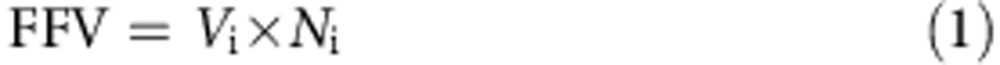


where *N*_i_ is the number density of cavities of type i. *N*_i_ was estimated based on the relationship


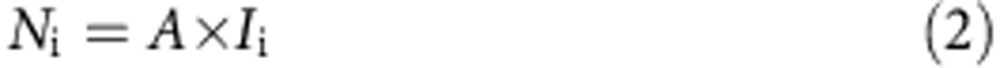


where *A* is an empirical constant equal to 0.0018, and *I*_i_ is the PALS intensity corresponding to cavities of type i (the ordinate in Fig. 4a). This is a well-established approach for characterizing free-volume in polymeric materials^23^,^24^. The free-volume determined by this approach is consistent with equation-of-state predictions for a variety of polymers^25^,^26^.

It should be noted that equation (2) is not appropriate for certain cases where electron scavenging is present. The absence of electron scavenging in our experiments was confirmed by two observations. First, the *o*-Ps intensities measured in our experiments range between 8 and 30%. Electron scavenging generally results in low-intensity values, usually 1–2%. Second, there was no decay in measured intensity as a function of time. We collected multiple data sets for each sample to ensure that both the lifetimes and intensities did not change with time. Time-dependent signals are a common signature of electron scavenging.

The fractional free-volume of EDE129-41 was found to be 13.9%. The EDE block copolymer contains two types of microdomains with very different free-volume characteristics. To account for contributions from the polyethylene-rich microdomains, we synthesized a polyethylene, PE homopolymer using the same protocol that was used for the synthesis of the triblock copolymers, and measured its free-volume by PALS. The *I*_i_ versus *d*_i_ curve thus obtained is given in the Supplementary Information (Supplementary Fig. 2). FFV of pure PE was determined to be 5.4%. The FFV of PDMS-rich microphases in our samples was estimated using the following equation:


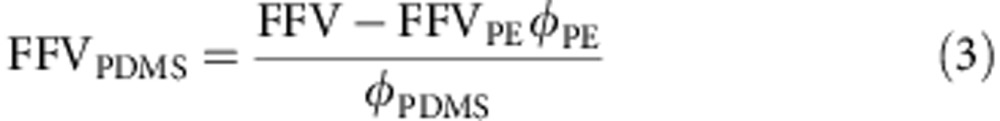


Equation (3) assumes that the PE-rich microphase in EDE contains the same fractional free-volume as the PE homopolymer. Polyethylene is a semicrystalline polymer, and it is generally assumed that the free-volume elements reside primarily in the amorphous regions. We measured the enthalpy of melting of all of our samples by differential scanning calorimetry (DSC) as described in the methods section. The PE homopolymer, the neat EDE129-41, and samples with artificial free-volume all showed percent crystallinities of about 28%. Based on Equation (3), FFV of the PDMS-rich microphase in EDE129-41 is 26.7 %.

The PALS data from samples with artificial free-volume, EDE129-41/9 and EDE129-41/17, revealed two populations, as shown in Fig. 4a. The intensity of the population with large cavities in these samples is significantly larger than that of the neat EDE129-41 sample. PALS analysis described above was performed on the samples with artificial free-volume. This analysis indicates that FFV_PDMS_ of EDE129-41/9 is 31.2 % while that of EDE129-41/17 is 33.9 %. These values are significantly higher than that obtained from neat EDE129-41 (26.7 %).

**Figure 5:**
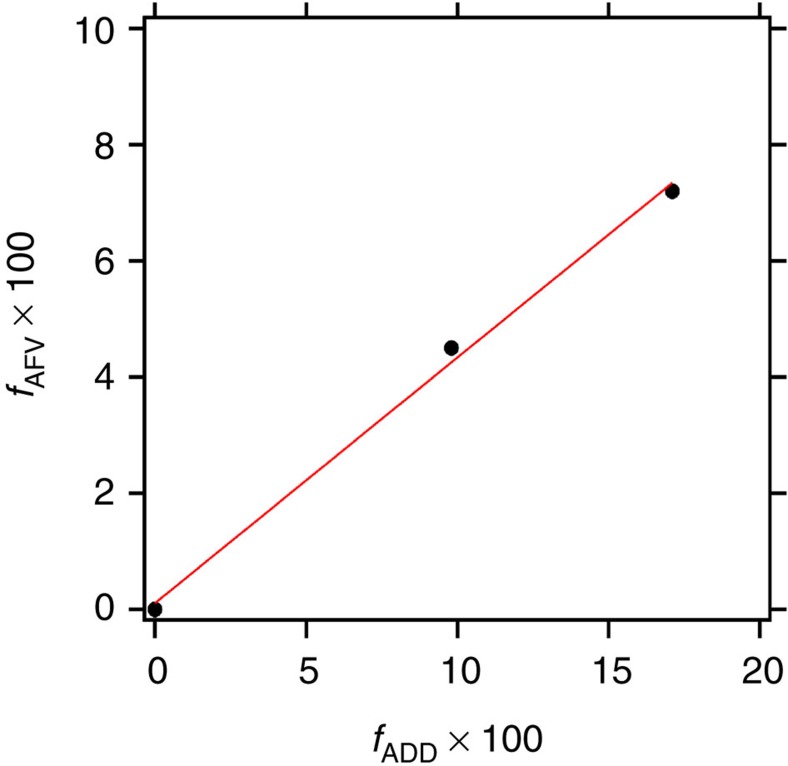
Relationship between theoretical free-volume, *f*_ADD_ and experimentally measured free-volume, *f*_AFV_. A linear fit of the data through the origin indicates that *f*_AFV_=0.44 × *f*_ADD_, indicating that 44% of the volume originally occupied by the PDMS polymer chains is converted into artificial free-volume. These results correspond to the EDE129-41 series of polymers.

The efficacy of our approach for creating artificial free-volume is quantified in Fig. 5. Here we plot the artificial fractional free-volume obtained by self-assembly, *f*_AFV_, versus the fraction of the PDMS microdomains occupied by PDMS homopolymer, *f*_ADD_. A linear fit of the data through the origin indicates that *f*_AFV_=0.44 × *f*_ADD_, indicating that roughly half amount of the volume originally occupied by the PDMS polymer chains is converted into artificial free-volume. We have thus accomplished our goal of increasing the free-volume of a polymeric phase without alteration of any other chemical characteristic of that phase.

It is instructive to compare the fractional free-volume achieved by our methodology with cross-linked PDMS, and other microporous materials. Literature values of free-volume for cross-linked PDMS range from 15 to 25 % depending on cross-linking density and method of cross-linking. Polymers with intrinsic microporosity and disubstituted polyacetylenes typically contain FFV in the range of 25–35% (these values correspond to free-volume of these materials before aging). The highest value of FFV_PDMS_ obtained in our study compares favourably with the values obtained in polymers with intrinsic microporosities. The overall FFV in our films is somewhat lower due to the presence of the polyethylene microphase. It is, however, conceivable that a systematic study of blended EDE block copolymers with artificial free-volume will enable the design of membranes with significantly higher free-volume than that reported here.

### Effects of artificial free-volume on permeation

It has been proposed that membrane separation techniques like pervaporation and vapour permeation can be coupled with a fermentation reactor for biofuel production. Such a process enables continuous removal of poisonous products resulting in longer fermentation times and higher sugar conversion. In this case, low-molecular weight alcohols (typically butanol and ethanol) have to be selectively permeated while water, the medium of the fermentation process, must be rejected by the separation membrane. High-membrane permeability and selectivity are important traits for cost-effective biofuel purification. Since biofuel molecules are larger than water, hydrophobic membranes with reverse selectivity are required to achieve high permeability and selectivity^27^. We thus tested our reverse-selective membranes with artificial free-volume for purifying two model mixtures relevant to biofuel production, butanol/water (1.5 wt. % butanol) and ethanol/water (8 wt. % ethanol) by pervaporation. We expect permeability through microphase separated block copolymer membranes to be proportional to the volume fraction of the transporting phase. Therefore butanol, ethanol and water permeabilities (*P*_B_, *P*_E_ and *P*_W_) through our membranes can be expressed as


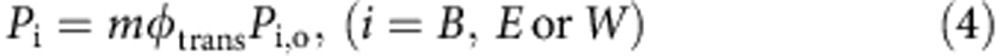


where *φ*_trans_ accounts for the different volume fractions of the PDMS-rich transporting phase that includes the volume fraction of the added homopolymer (*f*_ADD_) in each block copolymer, *P*_i,o_ is the intrinsic permeability of the pure transporting phase, and *m* is a morphology factor that accounts for geometric constraints on diffusion^28^. Equation (4) assumes that transport occurs exclusively in one of the microphases. For lamellar systems *m*=2/3 because, on average, one third of the lamellar grains will be oriented perpendicular to the direction of transport (all of our membranes have a lamellar morphology)^29^. The measured pervaporation data and our approach for determining permeabilities are described in the methods section. Since *P*_i_ is measured and *m* and *φ*_PDMS_ are known, we can use equation (4) to determine the intrinsic permeability of the transporting phase in our membranes, *P*_B,o_, *P*_E,o_, *P*_W,o_. The intrinsic butanol and ethanol permeabilities for the neat EDE samples were *P*_E,o_=8.5 × 10^12^ and *P*_B,o_=26 × 10^12^ mol m m^−2^ s^−1^ Pa^−1^. These values are higher than typical literature values for cross-linked PDMS (*P*_E_=6.0 × 10^12^ mol m m^−2^ s^−1^ Pa^−1^ and *P*_B_=21 × 10^12^ mol m m^−2^ s^−1^ Pa^−1^)^29^.

**Figure 6:**
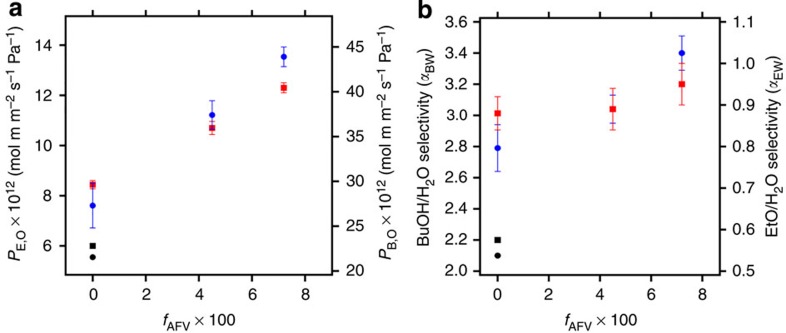
Membrane transport performance for butanol and ethanol. (**a**) Normalized butanol permeability (right ordinate) and normalized ethanol permeability (left ordinate) as a function of artificial free-volume, *f*_AFV,_ (bottom ordinate) for the series of membranes based on polymer EDE129-41. Blue circles: butanol permeability through EDE membranes; red squares: ethanol permeability through EDE membranes; black circle: butanol permeability by cross-linked PDMS membrane; and black square: ethanol permeability by cross-linked PDMS membrane. (**b**) Butanol/water (left ordinate) and ethanol/water (right ordinate) selectivity as a function of artificial free-volume *f*_AFV_ (bottom ordinate). Blue circles: butanol/water selectivity by EDE membranes; red squares: ethanol/water selectivity by EDE membranes; black circle: butanol/water selectivity by cross-linked PDMS membrane; and black square: ethanol/water selectivity by cross-linked PDMS membrane. Error bars represent the second s.d. of triplicated pervaporation experiments.

Figure 6a shows the dependence of *P*_E,o_ (left ordinate) and *P*_B,o_ (right ordinate) on *f*_AFV_. The introduction of artificial free-volume results in a significant increase in butanol and ethanol permeabilities as seen in Fig. 6a. The abscissa at the bottom of Fig. 6a has been used to quantify the effect of artificial free-volume on intrinsic butanol and ethanol permeabilities. Membrane EDE129-41/17 showed ethanol permeability of 12.1 × 10^12^ an increase of 43% and butanol permeability of 43.8 × 10^12^ an increase of 68% compared with neat EDE samples.

Butanol and ethanol permeabilities through EDE129-41/9 and EDE129-41/17 were measured 2 months after the experiments reported in Fig. 6 were completed. These permeabilities were within experimental error of those reported in Fig. 6. This demonstrates the stability of artificial free-volume created by block copolymer self-assembly.

The efficacy of a reverse-selective membrane is determined by both absolute flux and selectivity, α_i,w_ (i=B or E)


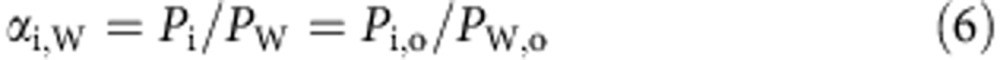


The effect of artificial free-volume on selectivity is shown on Fig. 6b where α_B,W_ and α_E,W_ are plotted versus *f*_AFV_.

It is evident from Fig. 6b that the enhancement in permeability reported in Fig. 6a is not obtained at the expense of selectivity. In fact, selectivity increases slightly with increasing *f*_AFV_. The data in Fig. 6b suggest that the artificial free-volume created by the self-assembly process is more hydrophobic than that present in cross-linked PDMS. The dielectric constant of vacuum (8.85 × 10^−12^ F m^−1^) is lower than that of PDMS (2.2 × 10^−11^ F m^−1^). One thus expects that transport of hydrophobic molecules such as butanol to be enhanced by artificial free-volume relative to that of polar molecules such as ethanol and water. The data in Fig. 6a and b are consistent with this expectation. The performance of EDE membranes with artificial free-volume is thus not subject to the typical trade-off of flux versus selectivity^11^,^30^,^31^.

## Discussion

In summary, we have demonstrated that it is possible to increase the size and concentration of free-volume cavities in a controlled fashion by block copolymer self-assembly. We thus obtain membranes that are chemically identical to their precursors but contain systematically varied levels of free-volume. The utility of these materials was demonstrated by using them to selectively remove alcohols from butanol/water and ethanol/water mixtures. These separations are relevant to biofuel production. The presence of artificial free-volume resulted in increase of both butanol and ethanol permeabilities without adversely affecting selectivity. We anticipate that this methodology can be useful for synthesizing wide range of polymeric materials with controlled amount of free-volume and improved performance for technologically important applications.

## Methods

### Pervaporation experiments

Pervaporation experiments of ethanol/water and butanol/water mixtures were conducted on a laboratory bench test unit built by Sulzer Chemtech, Germany. The membrane was held inside a circular cell restrained with an o-ring, providing a total permeation area of 37 cm^2^. The temperature of the feed was controlled in the range of 40±1 °C. Each experiment began with ∼2 l of 8 wt % ethanol/water solution or 1.5 wt % butanol/water in the feed tank. On the permeate side of the membrane, a vacuum of 2–3 mbar was applied using a vacuum pump (Welch, model 2014) and permeates were condensed in a trap cooled with a dry-ice/isopropanol mixture at −80 °C. After starting the feed pump, the system was allowed to attain steady state for 1 h before permeate samples were collected. For each polymer, two different membranes were prepared and pervaporation experiments were repeated twice for each membrane. The average values of the four runs are reported and the s.d. is taken to be the uncertainty of the measurements.

Permeate samples were weighed to determine the mass permeated through the membrane during the experiment. The feed composition was fixed at 8 wt % for ethanol and 1.5 wt % for butanol. Changes in feed composition due to pervaporation are negligible due to small amounts permeating through the membrane. Flux of water, ethanol or butanol was calculated using the equation,


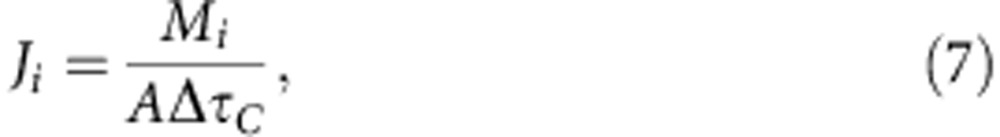


where *M*_i_ is the mass of individual permeant, *A* is the permeation area (37 cm^2^) and Δ*τ*_C_ is the permeate collection time; subscript E implies ethanol, B implies butanol while subscript W implies water. Membrane permeability, *P*_i_, was calculated from the following equation,


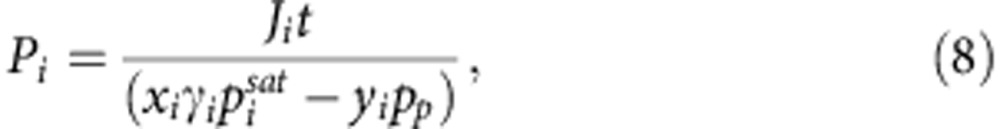


where *t* is the membrane thickness, *x*_i_ is the feed mole fraction, *γ*_i_ is the activity coefficient, 
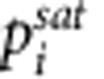
 is the saturated vapour pressure, *y*_i_ is the permeate mole fraction and *p*_p_ is the permeate pressure. Values of *y*_i_ were determined by analyzing permeate samples by ^1^H NMR spectroscopy with deuterated acetone (acetone-d_6_) as the solvent. The activity coefficients were calculated using the Van Laar coefficients and the saturated vapour pressure 
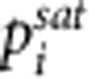
 was determined using the Antoine equation.

We use units of mol m m^−2^ s^−1^ Pa^−1^ in this paper when we report *P*_E_ or *P*_W_.

### Positron annihilation lifetime spectroscopy

PALS was used to determine the free-volume within the membranes by measuring the lifetime of positrons within the polymer membranes before they annihilate due to interactions with the material. Positrons form a bound state with free electrons within the membranes with the same spin state (*o*-Ps). The *o*-Ps is attracted to areas of low electron density (free-volume) and annihilate when interacting with electrons from the membrane. Therefore, a relationship between the size of the free-volume elements within the sample can be made with the lifetime of the *o*-Ps. The longer the lifetimes, the larger the free-volume within the material. The Tao-Eldrup equation is used to calculate the average free-volume using the *o*-Ps lifetime (*τ*_3_);^32^,^33^


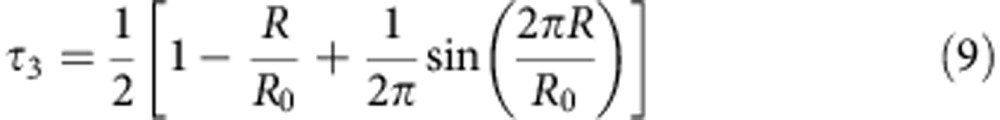


The semi-empirical equation assumes an infinite spherical potential well model where R is the radius of the free-volume elements and R_0_=R + ΔR (where ΔR was calculated to be 1.66 Å due to the thickness of the electron layer within the potential well of radius R_0_).

The FFV was calculated assuming spherical free-volume elements using the radius determined from the lifetime (R) and the associated Intensity (I_3_)^34^.


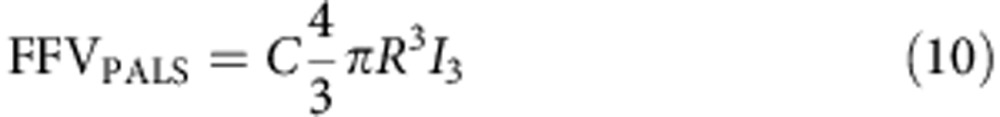


Here, *C* is an empirical constant determined to be ∼0.0018, nm^−3^.

The membranes were measured on an EG&G Ortec fast–fast coincidence system using ^22^NaCl (∼1.5 × 10^6^ Bq) as the source of positrons which was sealed in a Mylar envelope. The membranes were cut and stacked into 2 mm thick bundles and placed either side of the positron source. The measurements were taken under vacuum (1 × 10^−5^ torr) with a minimum of five files collected at 4.5 × 10^6^ integrated counts per file for each membrane. A source correction of 1.48 ns and 3.033% was subtracted from each spectra. The spectra were deconvoluted using LT v.9 software^35^. Each spectrum was fitted to four components with the first two components fixed to 0.125 ns (Para-positonium, due to a bound state of opposite spin) and approximated to 0.4 ns (free annihilation). The third and fourth component was due to *o*-Ps annihilation events indicating the presence of two distinct pore sizes within the membranes.

### NMR spectroscopy

^1^H NMR measurement was conducted on 500 MHz Bruker DRX 500 spectrometer using deuterated solvents purchased by Aldrich. The solutions for ^1^H NMR spectra had a polymer concentration of ∼10 mg ml^−1^. Polymers post hydrogenation were analysed at 80 °C to ensure complete solubilization of the polyethylene segments. Spectra were analysed to determine copolymer compositions as well as hydrogenation and homopolymer removal percentages.

### Scanning transmission electron microscopy

Thin sections with thickness of ∼120 nm were obtained by cryo-microtoming at −120 °C using a Leica EM FC6 and picked up on a lacey carbon coated copper grid (Electron Microscopy Sciences). Scanning transmission electron microscopy experiments were performed on a Tecnai F20 UT FEG, equipped with a high-angle annular dark-field detector, using 200 keV acceleration voltage.

### Differential scanning calorimetry

DSC experiments were performed on a Thermal Advantage Q200 calorimeter. Samples were sealed in aluminium hermetic pans. DSC scans consisted of two heating/cooling cycles and were conducted over the range 0–150 °C at a rate of 10 °C min^−1^. The enthalpy of fusion of the first heating cycle was used in order to estimate the degree of crystallinity of polyethylene and the polyethylene phase in the EDE triblock copolymer samples by comparing it with the enthalpy of fusion of 100% crystalline polyethylene. A value of 4.11 kJ per repeating unit was used to estimate the enthalpy of fusion of 100% crystalline polyethylene.

### Gel permeation chromatography

*M*_n_ and dispersities, *D*, of the polybutadiene-*b*-polydimethylsiloxane-*b*-polybutadiene (BDB) precursors were obtained using a Viscotek TDA 302 gel permeation chromatography system that has a guard column, a set of four Viscotek columns (300 mm × 7.8 mm, T-3,000, T-4,000, T-5,000 and T-6,000 columns) and a refractive index detector, with THF eluent (flow rate of 1 ml min^−1^, 35 °C). The instrument was calibrated with polystyrene standards (Agilent Easivials PS-M). The molecular weights of the poly(1,4-butadiene) precursors were calculated based on triple detection experiments.

### Synthesis of EDE

All manipulations were carried out by standard high-vacuum techniques and glove box operations, unless otherwise stated. sec-BuLi (Aldrich 1.7 M in cyclohexane) was titrated before use by the 1,3-diphenylacetone p-tosylhydrazone method to confirm the precise concentration of active species and it was used without any additional treatment. THF was purified by passing through solvent purification columns followed by stirring overnight over finely ground CaH_2_ and then stored in a reactor over a Na/benzophenone mixture obtaining the characteristic blue colour. Toluene was purified by passing through solvent purification columns followed by stirring overnight over finely ground CaH_2_. Finally it was distilled and stored in a reactor over polystyryl lithium obtaining the characteristic red colour. Solvents were distilled on the vacuum line before use. The purification of hexamethylcyclotrisiloxane, D_3_, (gelest 95%) was performed as follows: The appropriate amount of D_3_ was melted by heating at 80 °C, put in a flask, diluted by an equal amount of purified cyclohexane and stirred overnight over CaH_2_. Then the solvent along with the monomer was distilled into a flask containing polystyryl lithium. The monomer was in contact with polystyryl lithium for about 2 h at room temperature and then it was distilled into a flame-dried reactor along with the solvent by heating at 80 °C. Finally, the monomer was isolated by distilling cyclohexane to another reactor at room temperature. The reactor containing D_3_ was transferred and stored in the glove box. The purification of 1,3-butadiene (Aldrich, 99%) was performed by drying an appropriate amount of monomer over finely ground CaH_2_ overnight, followed by distillation in activated molecular sieves where it remained in contact overnight. Then butadiene was vacuum transferred to a reactor containing n-BuLi where it remained with continuous stirring at 0 °C for 1 h. The monomer was distilled in a reactor containing toluene that had been dried as mentioned previously. The monomer was stored at −20 °C in the glove box and was used within the period of 2 weeks. The linking agent 1,2-bis-(dimethylchlorosilyl)ethane (Aldrich) was purified by fractional distillation on the vacuum line and then stored in the glove box.

As an example of a polymerization experiment the synthesis of BDB335-78 has as follows: 100 ml of cyclohexane and 10 ml of 1,3-butadiene (0.114 mol), that had been purified and stored, as described in the previous section, were vacuum transferred in a Schlenk reactor. Subsequently the Schlenk reactor containing butadiene and cyclohexane was transferred in the glove box where 0.02 ml of sec-BuLi (0.000213, mol) were added (target molecular weight 30 k). The reactor containing solvent, monomer and initiator was immediately placed in an oil bath preheated at 50 °C. The reaction was allowed to proceed for 18 h. Then the reactor was transferred in the glove box and a sample was removed. The sample was quenched by introducing an excess of methanol and was used for characterization purposes (^1^H NMR spectroscopy and gel permeation chromatography). Then 26.6 g of purified D_3_ (0.120 mol) were introduced (target molecular weight 125 k) and the Schlenk reactor was left in contact with the living polybutadiene anions overnight. The THF (dried as mentioned in the previous section) was returned on the vacuum line, and 100 ml were vacuum transferred in a graduated ampule. The ampule was transferred in the glove box and the THF was added in the polymerization reactor (cyclohexane/THF: 50% v/v). The reactor was left at room temperature for 90 min and then was removed from the glove box and emerged in a chiller with a temperature pre-set at −20 °C. The polymerization was left for 72 h and then the reactor was transferred in the glove box where 0.046 g of purified 1,2-bis(dimethylchlorosilyl)ethane (0.000213, mol) were introduced. The coupling reaction was left for 45 min and then excess of trimethylchlorosilane was added. The polymer was precipitated six times in methanol and dried under vacuum. Finally, for long-term storage, the polymer was dissolved in cyclohexane and butyl hydroxyl toluene was added as an inhibitor. Then cyclohexane was removed under vacuum and the polymer/butyl hydroxyl toluene mixture was stored at −20 °C. The volume fractions of the PDMS block of the BDB and EDE copolymers (*φ*_PDMS_) were estimated using monomer volumes of 0.111, 0.138 and 0.119 nm^3^ for polybutadiene, PDMS and PE, respectively.

Hydrogenation of BDB copolymers was carried out in a 1 l three-neck round bottom flask which was equipped with magnetic stirring, a reflux condenser, a thermometer and a stopper. The apparatus was supplied with positive pressure of dry argon. Predetermined amounts of BDB block copolymer and *o*-xylene were added, and the mixture was left to stir for 1 h at 60 °C. This resulted in the complete dissolution of the polymer. Predetermined amounts of *p*-toluenesulfonyl hydrazide and tripropylamine were added to the flask and the temperature was raised to the desired set point. The reaction was quenched by precipitating the product in ice cold methanol.

## Additional information

**How to cite this article:** Petzetakis, N. *et al.* Membranes with artificial free-volume for biofuel production. *Nat. Commun.* 6:7529 doi: 10.1038/ncomms8529 (2015).

## Supplementary Material

Supplementary Information Supplementary Figures 1-2

## Figures and Tables

**Table 1:** Characteristics of polymer membranes studied in the present work.

**Membrane**	*M_**n**_^**exp**^ **(kg mol**^**−1**^)	†***φ***_**PDMS**_	***r***	‡**Morphology**	§***d***_**EDE**_ **(nm)**	||***f***_**ADD**_
EDE129-41	38-54-38	0.41	−	Lamellar	17.4	−
EDE129-41/9	38-54-38	0.45	0.26	Lamellar	18.5	9
EDE129-41/17	38-54-38	0.48	0.26	Lamellar	19.7	17
EDE209-45	53-102-53	0.45	−	Lamellar	32.2	−
EDE209-45/7	53-102-53	0.48	0.14	Lamellar	33.5	7
EDE209-45/28	53-102-53	0.58	0.14	Lamellar	34.9	28

r is the ratio of the molecular weight of the PDMS homopolymer to that of the PDMS block in the copolymer

^*^Molecular weights obtained by combination of gel permeation chromatography and ^1^H NMR spectroscopy.

^†^PDMS volume fraction in the neat and composite membranes.

^‡^Morphologies obtained by small angle X-ray scattering and transmission electron microscopy.

^§^Domain spacing obtained by small angle X-ray scattering.

^||^Volume fraction of PDMS homopolymer in the PDMS microphase of the composite membranes multiplied by 100.
