# Supplementary material for: Membranes with artificial free-volume for biofuel production
Source: Nat Commun. 2015 Jun 24;6:7529. doi: 10.1038/ncomms8529 (PMC4491837; doi:10.1038/ncomms8529)
Supplement: Supplementary Information — Supplementary Figures 1-2 [file ncomms8529-s1.pdf]

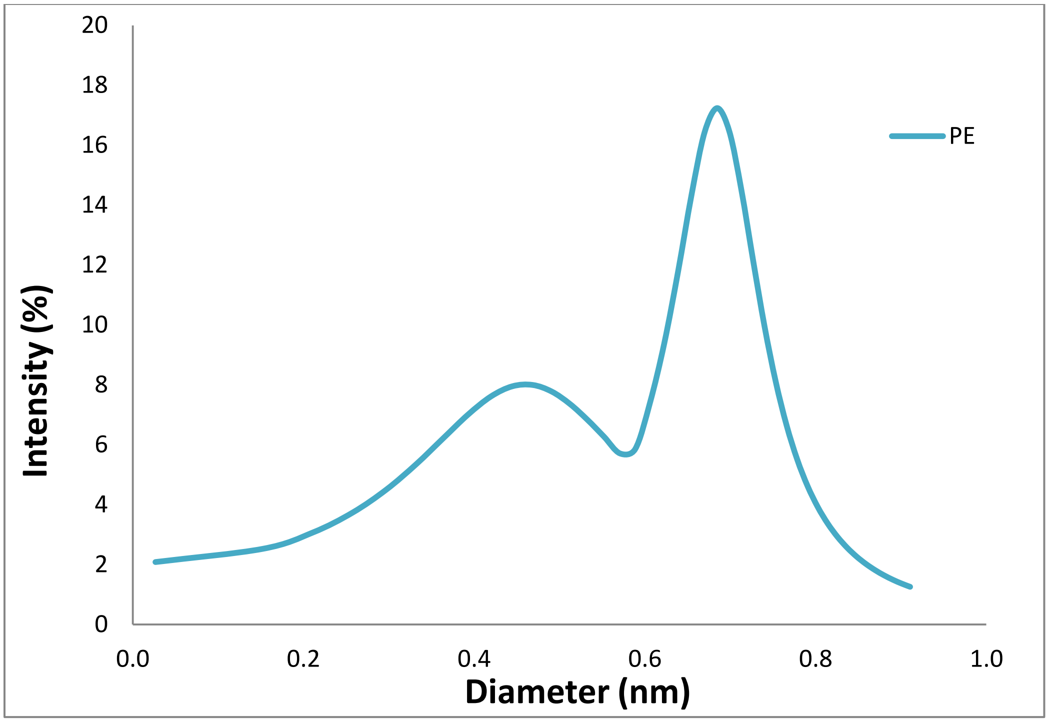


**Supplementary Figure 1. PALS free-volume cavity distributions obtained by polyethylene homopolymer.** Data used to calculate the fractional free-volume in the PDMS microphase.

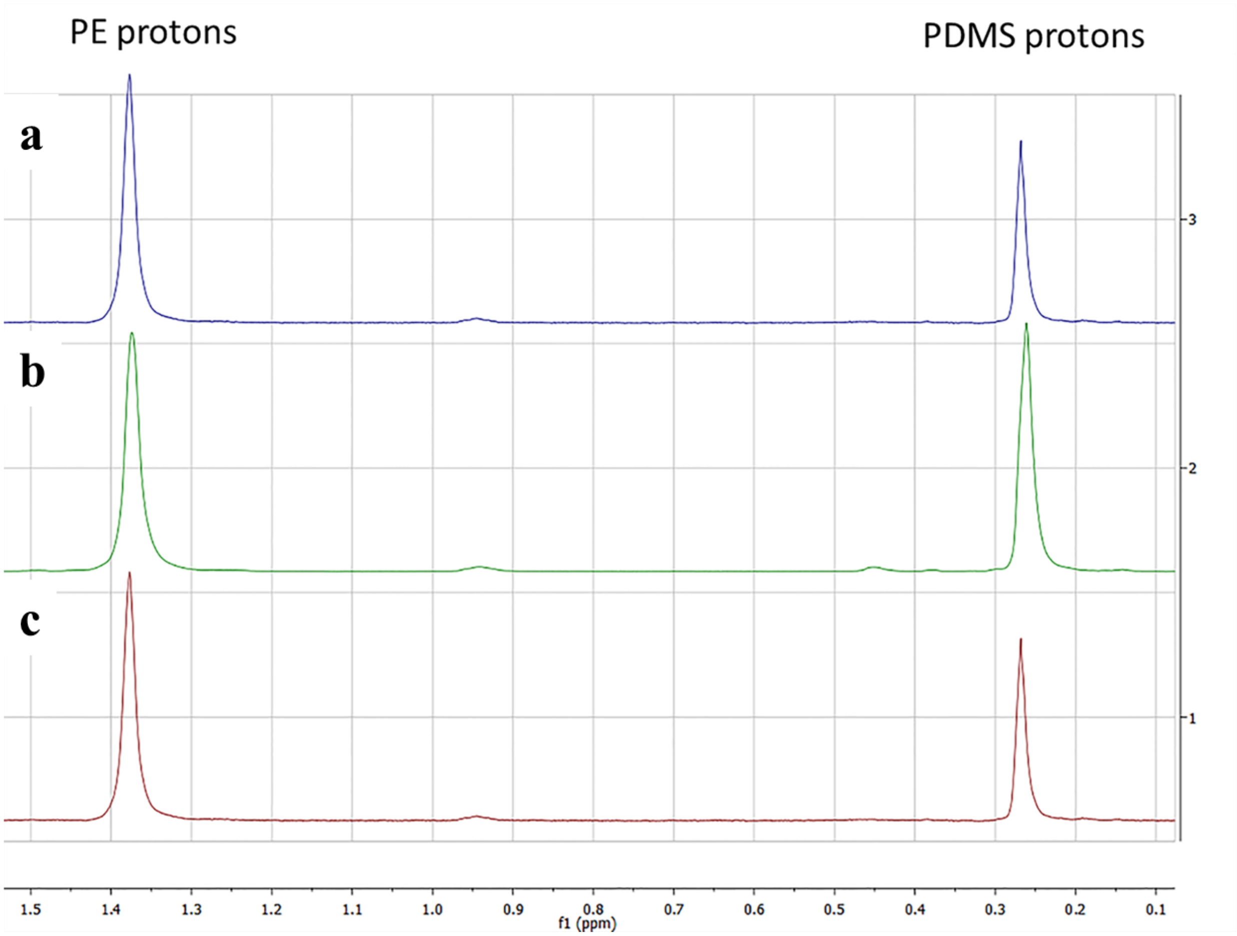


**Suplemmentary Figure 2. $^{1}$H NMR data.** a) pristine block copolymer EDE129-41, b) the block copolymer with added homopolymer and c) the block copolymer after removal of the homopolymer (EDE129-41/9).
